# Casein- and Soy-Based High-Protein Diets Differentially Affect Insulin Resistance and Adipose Tissue Advanced Glycation End Product Accumulation in Obese Diabetic Mice

**DOI:** 10.1016/j.cdnut.2026.107647

**Published:** 2026-01-27

**Authors:** Yoshie Shiraga, Yusaku Mori, Naoya Osaka, Michishige Terasaki, Hironori Yashima, Tomomi Saito, Daiki Tanno, Madoka Ogino, Makoto Ohara, Sho-ichi Yamagishi

**Affiliations:** 1 Department of Diabetes, Metabolism, and Endocrinology, Showa Medical University Graduate School of Medicine, Tokyo, Japan; 2 Anti-glycation Research Unit, Division of Medicine, Showa Medical University Graduate School of Medicine, Tokyo, Japan

**Keywords:** advanced glycation end products, high-protein diet, insulin resistance, visceral adipose tissue, diabetes

## Abstract

**Background:**

Replacing dietary carbohydrates with protein has been proposed as a nutritional strategy to improve glycemic control and reduce obesity in individuals with type 2 diabetes. However, high-protein diets (HPDs) may also facilitate the formation of advanced glycation end products (AGEs), pathogenic molecules associated with insulin resistance and various diabetic complications.

**Objectives:**

This study investigated the effects of animal- and plant-based high-protein, low-carbohydrate diets on insulin resistance and tissue AGE accumulation in obese diabetic mice.

**Methods:**

Male KK-Ay mice were fed either a standard diet (STD; 64% carbohydrate and 20% casein of total energy) or casein- and soy-based HPDs (casein-HPD and soy-HPD; 43% carbohydrate and 41% casein or soy protein of total energy, respectively) for 12 wk. Blood, urine, epididymal adipose tissue, and kidneys were collected for biochemical, histological, and molecular analyses.

**Results:**

Compared with the STD, the casein-HPD reduced glycated hemoglobin concentrations (12.4% compared with 9.9%) without affecting body weight gain or energy intake, but it significantly exacerbated insulin resistance (467% increase compared with STD). In epididymal adipose tissue, the casein-HPD–induced marked accumulation of glyceraldehyde-derived AGEs (glycer-AGEs), a highly toxic subtype, accompanied by increased oxidative stress, macrophage infiltration, and reduced adiponectin expression. The casein-HPD also modestly impaired renal function and increased renal glycer-AGE and oxidative stress concentrations without affecting proteinuria or structural changes. In contrast, the soy-HPD did not alter glycated hemoglobin, insulin resistance, renal function, or tissue AGE accumulation. All diets contained negligible glycer-AGE concentrations, indicating that endogenous formation was selectively enhanced by the casein-HPD.

**Conclusions:**

A casein-HPD was associated with greater insulin resistance in this model, concurrent with increased glycer-AGE accumulation in visceral adipose tissue, whereas the soy-HPD did not result in substantial differences compared with the STD. These observations suggest that the metabolic effects of HPDs may differ depending on the protein source.

## Introduction

The prevalence of type 2 diabetes has been rising worldwide [1], and its complications, including retinopathy and nephropathy, are now estimated to affect >530 million people [1]. The main pathogenesis of type 2 diabetes involves increased insulin resistance, primarily due to visceral fat accumulation, together with inadequate compensatory insulin secretion from pancreatic *β*-cells, which leads to chronic hyperglycemia [2,3]. Although many pharmacological agents, such as glucagon-like peptide-1 receptor agonists, have recently been developed and their efficacy for reducing hyperglycemia and body weight is widely proven, their use is limited by accessibility, affordability, and side effects [3,4]. Consequently, dietary interventions aimed at reducing body fat, particularly visceral fat deposition, remain a fundamental strategy for both the prevention and management of type 2 diabetes [5].

Among nutrients, carbohydrates are the major contributor to postprandial hyperglycemia, and replacing part of dietary carbohydrates with protein has been proposed as a possible beneficial strategy for improving glycemic control in individuals with type 2 diabetes [6,7]. Indeed, numerous preclinical and clinical studies have demonstrated that high-protein diets (HPDs) improve hyperglycemia and obesity compared with isocaloric high-carbohydrate diets [6,7]. However, long-standing concerns remain regarding the potential adverse effects of excessive protein intake on renal function, particularly in individuals with kidney disease such as diabetic nephropathy [[8], [9], [10], [11]]. In addition, some studies have suggested that HPDs may exacerbate insulin resistance under certain conditions [12,13]. Conversely, accumulating evidence has indicated that plant-based proteins may exert less deleterious effects on insulin resistance and renal injury than animal-based proteins [[14], [15], [16]]. More recently, soy protein has been extensively studied as a representative plant-based protein and has been reported to exert beneficial effects on glucose metabolism and insulin sensitivity in both human studies and rodent models [17]. Clinical studies have suggested that soy protein intake may improve glycemic control compared with animal-based proteins [18], whereas experimental studies have demonstrated its anti-inflammatory and insulin-sensitizing properties in animal models [19]. Nevertheless, the precise molecular mechanisms underlying the differential effects between animal-based and plant-based protein diets have not yet been fully elucidated.

Advanced glycation end products (AGEs) are senescent macromolecular derivatives formed through nonenzymatic reactions between monosaccharides and amino groups of proteins, lipids, or nucleic acids [20], and their formation and accumulation are markedly accelerated under diabetic conditions. Accumulating evidence indicates that AGEs could induce oxidative stress and inflammatory responses in various tissues and organs through interactions with their cell-surface receptor, the receptor for AGEs (RAGE), thereby contributing to the development and progression of insulin resistance and various diabetic complications [21]. Because proteins are major substrates for AGE formation, there is concern that high-protein intake may promote AGE generation and subsequent accumulation. However, it remains unclear whether HPDs—particularly those derived from animal sources—induce AGE accumulation and whether such accumulation is associated with the deleterious effects of HPDs in individuals with type 2 diabetes.

AGEs comprise a heterogeneous group of molecular species with distinct chemical and biological properties [22]. We previously demonstrated that glyceraldehyde-derived AGEs (glycer-AGEs) possess high cytotoxicity with a strong binding affinity for RAGE [22]. Furthermore, inhibition of the glycer-AGEs/RAGE axis by DNA aptamers effectively prevented the progression of diabetic nephropathy and insulin resistance in animal models of diabetes [[23], [24], [25]]. Moreover, circulating concentrations of glycer-AGEs have been shown to correlate with insulin resistance in nondiabetic subjects [26]. These observations suggest that glycer-AGEs play a central role in mediating the pro-oxidative and proinflammatory effects of AGEs in diabetes [21], thereby leading us to hypothesize that animal-based high-protein intake would exacerbate insulin resistance and renal injury by inducing glycer-AGE accumulation in the adipose and renal tissues compared with plant-based high-protein intake. In the present study, we examined whether and how animal- and plant-based HPDs influence systemic metabolic parameters and glycer-AGE accumulation, as well as adipose inflammation and kidney injury, in KK-Ay mice, a well-established animal model exhibiting key characteristics of human type 2 diabetes with obesity [27,28].

## Methods

### Animal experiments

The study protocol was approved and registered by the Animal Care Committee of Showa Medical University School of Medicine (approval number: 125004). Animal experiments were conducted in accordance with modified Animal Research: Reporting of In Vivo Experiments (ARRIVE) 2.0 guidelines and the Guide for the Care and Use of Laboratory Animals (8th edition) [29,30]. All invasive procedures were performed under general anesthesia with isoflurane, except for blood collection for plasma glucose and glycated hemoglobin (HbA1c) measurements. Six-week-old male KK-Ay mice (*n* = 18) were purchased from Nihon Clea. Sample size calculation is described in the statistical analysis section. Animals were housed individually in standard cages and provided standard chow (Labo MR Stock, NOSAN) with ad libitum access to food and water. They were maintained in a specific pathogen-free facility at the Division of Animal Experimentation, Showa Medical University School of Medicine, under controlled conditions (12 h light/dark cycle, 21°C, and 40%–60% relative humidity).

After a 2-wk acclimatization period, mice were randomly assigned to one of the following isocaloric diets with varying protein amounts or sources according to cage number by an independent researcher (YM) (*n* = 6 per group): *1*) standard diet (STD; total energy: 64% carbohydrate, 20% protein from casein, 16% fat), *2*) casein-based high-protein, low-carbohydrate diet (casein-HPD; total energy: 43% carbohydrate, 41% protein from casein, 16% fat), or *3*) soy-based high-protein, low-carbohydrate diet (soy-HPD; total energy: 43% carbohydrate, 41% protein from soy, 16% fat) [10,11,31]. Casein and soy were selected because they are well-standardized protein sources widely used in nutritional studies, facilitating comparison with previous work. Casein represents a high-quality animal protein, whereas soy provides a plant-based protein with a distinct amino acid profile [10,11,31]. A protein concentration of ∼40% is a commonly used definition of a HPD in rodent studies and provides sufficient contrast to detect protein-dependent metabolic changes [10,11,32]. Although this protein concentration exceeds typical human intakes—generally around 15% to 20% of energy and ∼25% to 30% in HPDs—it remains within the standard range used for mechanistic rodent studies [32]. All diets were prepared to be isocaloric and matched for total energy density, and were obtained from Research Diets. Detailed compositions of each diet are provided in Supplemental Table 1. Each mouse’s health and body weight were monitored weekly during the intervention. Food intake was measured by housing each mouse individually and weighing the remaining food once weekly. No unexpected adverse event was observed during the experimental period. After 12 wk of dietary intervention, urine samples were collected >12 h using individual metabolic cages, and blood samples were obtained via a small tail prick after a 6 h to 8 h fasting period for plasma glucose and HbA1c measurements. Subsequently, additional blood was collected from the inferior vena cava. The right epididymal fat pad and right kidney were harvested after perfusion fixation with 4% paraformaldehyde for histological and immunohistochemical analysis. Tissue processing and analyses were performed by blinded investigators (S-iY and TS). After sample collection, the mice were killed by an isoflurane overdose. All animals were included in the statistical analyses.

### Measurement of anthropometric and biochemical parameters

Plasma glucose concentrations were measured using an enzymatic electrode method (StatStrip 2; Nipro), and plasma and urinary protein, lipids, and creatinine concentrations were analyzed with enzymatic colorimetric assays (Pierce BCA Protein Assay Kit, Thermo Scientific Japan; Fujifilm Wako Pure Chemical Corporation; Serotec, respectively). Creatinine clearance was calculated using the following formula: creatinine clearance (μL/min) = urinary creatinine (mg/dL) × urinary volume (μL/min)/plasma creatinine (mg/dL). The urinary protein excretion rate was determined as the ratio of urinary protein concentration (μg/mL) to urinary creatinine concentration (mg/mL). Blood HbA1c concentrations were determined with an immunoassay (Roche Diagnostics Japan). Plasma insulin and glycer-AGE concentrations were quantified with ELISA (insulin: ultrasensitive mouse insulin measurement kit, Product number M1104, Morinaga; glycer-AGEs: rat polyclonal), as previously described [23]. HOMA-IR and *β*-cell function (HOMA-*β*) were calculated according to the equations of Matthews et al. [33] as follows: HOMA-IR = plasma glucose (mg/dL) × insulin (μU/m)/405, HOMA-*β* = plasma insulin (μU/m) × 360/[plasma glucose (mg/dL) − 63]. Blood pressure and pulse rates were measured on days 23 to 25 using a noninvasive tail-cuff method (Model MK-2000ST; Muromachi Kikai) [34]. The mean of 3 to 5 consecutive measurements was used as a single data point for each mouse.

### Morphometric analysis

After fixation in 4% paraformaldehyde for 24 h, epididymal fat pad and kidney samples were embedded into paraffin blocks. Tissue sections were obtained from the central portion of each sample and stained with hematoxylin and eosin for adipocyte size in epididymal fat pads, periodic acid–Schiff for evaluation of glomerular mesangial area, and Masson’s trichrome for medullary interstitial fibrosis in the kidney [23]. Stained sections were digitized using a confocal microscope (BZ-X710; Keyence) and analyzed with ImageJ software (NIH).

### Immunofluorescence staining

Immunofluorescence staining was performed as previously described [34]. Tissue sections were incubated overnight with one of the following primary antibodies: anti-glycer-AGEs (rat polyclonal; 1:100) [23], anti-8-hydroxy-2′-deoxyguanosine (8-OHdG) (product identification: MOG-020P; RRID: AB_1106819; mouse monoclonal; 1:200; Nikken Seil), and anti-F4/80 (product identification: Ab204467; RRID: AB_2810932; rat monoclonal; 1:250; Abcam Japan). Sections were then incubated with appropriate secondary antibodies for 4 h, and mounted with VECTASHIELD Antifade Mounting Medium (Vector Laboratories). Immunofluorescence images were captured using a confocal microscope (BZ-X710) and analyzed with ImageJ software.

### Real-time RT-PCR

Total RNA was extracted from tissues and used to synthesize complementary DNA for RT-PCR assay, as previously described [34]. Quantitative real-time RT-PCR was performed using the TaqMan Gene Expression Assay and sequence detection system (QuantStudio 3; Life Technologies Japan) [33]. The following predesigned TaqMan probe sets were used for the assay: *Adiponectin*, Mm04933656_m1; *monocyte chemotactic protein-1* (*Mcp-1)*, Mm00441242_m1; *Rage*, Mm00545815_m1; 18S ribosomal RNA (*18s rna*), Mm03928990_g1. Expression concentrations of each target gene were normalized to those of the internal control *18s rna*.

### Glycer-AGE measurement in diet samples

Each diet sample was powdered using microbeads, and 100 mg of the resulting powder was placed into 2.0 mL microtubes. Lipids were first removed by adding 0.4 mL of ice-cold acetone and incubating the mixture at −20°C for 30 min, followed by centrifugation at 10,000 × *g* for 10 min. After discarding the supernatant, the resulting pellet was dissolved in 1 mL of Tris–HCl buffer (pH 7.4) containing either 0.5% Triton X-100 or 0.1% SDS to extract proteins, and the mixture was incubated for 60 min. The sample was then centrifuged again at 10,000 × *g* for 10 min, and the supernatant was collected. Proteins were precipitated by adding 4 mL of ice-cold acetone and incubating at −20°C for 60 min, followed by centrifugation at 10,000 × *g* for 10 min. The resulting protein pellet was briefly air-dried to remove residual acetone and dissolved in 150 μL of phosphate-buffered saline (PBS). Glycer-AGEs prepared as described previously [23] were used as positive controls, and PBS was used as a negative control. Glycer-AGE concentrations in diet extracts were quantified using an ELISA, as described above [23].

### Statistical analysis

Data are presented as mean ± SD. Comparisons among groups were performed using 1-way analysis of variance (ANOVA) followed by Tukey’s post hoc test. For data involving repeated measurements, repeated-measures ANOVA with Bonferroni’s correction was applied. Correlations were evaluated using Pearson’s correlation coefficient. Statistical analyses were conducted using JMP Pro 17 software (SAS Institute). The required sample size for each group was estimated based on the following assumptions: comparison using 1-way ANOVA with Tukey’s post hoc test among 3 groups; type I error (*α*), 5%; type II error (*β*), 20% (power = 80%); an expected mean difference of 15%; and an expected within-group SD of 25%. The significance level was defined as *P* < 0.05.

## Results

### Effects of casein-HPD and soy-HPD on body weight, glycemic parameters, insulin resistance, and circulating glycer-AGEs in obese diabetic KK-Ay mice

**FIGURE 1:**
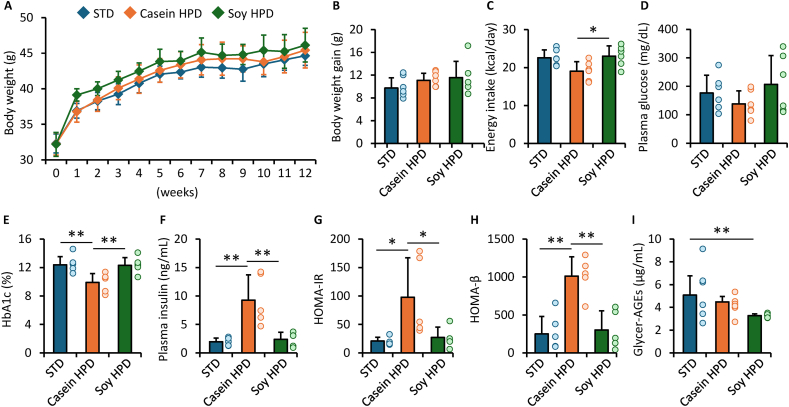
Effects of casein- and soy-based HPD on obesity, glycemic control, insulin resistance, and circulating glycer-AGEs in obese diabetic mice. (A) Body weight changes during the dietary intervention. (B) Overall body weight gain from baseline to week 12. (C) Energy intake during the dietary intervention. (D, E, F) Fasting plasma glucose, blood HbA1c, and plasma insulin concentrations at the end of the intervention. (G, H) HOMA-IR and HOMA-β at the end of the intervention. (I) Plasma AGE concentrations at the end of the intervention. (A, B, C, D, I) *n* = 6 per group. (F, G, H) STD, *n* = 6; casein-HPD and sou-HPD, *n* = 5. ∗*P* < 0.05, ∗∗*P* < 0.01. AGE, advanced glycation end product; Glycer-AGE, glyceraldehyde-derived AGE; HbA1c, glycated hemoglobin; HOMA-β, HOMA of β-cell function; HPD, high-protein diet; STD, standard diet.

Figure 1A shows body weight changes during the dietary intervention. Initial body weights were comparable among the 3 groups. Body weights at each time point were also comparable, and overall weight gain from baseline did not differ significantly among the groups (Figure 1B). Energy intake during the dietary intervention was significantly lower in the casein-HPD group than in the soy-HPD group (Figure 1C). At the end of the intervention, fasting plasma glucose concentrations did not differ among the 3 groups (Figure 1D). In contrast, HbA1c concentrations were significantly lower in the casein-HPD group than in both the STD and soy-HPD groups (Figure 1E). Plasma insulin concentrations, HOMA-IR, and HOMA-*β* were all significantly higher in the casein-HPD group than in the other 2 groups (Figure 1F–1H). Plasma glycer-AGE concentrations were significantly lower in the soy-HPD group than in the STD group (Figure 1I). In addition, systolic blood pressure, pulse rate, and plasma concentrations of total protein and lipids were comparable across the groups (Table 1).

**TABLE 1:** Anthropometric and biochemical parameters of obese diabetic mice after 12 wk of dietary intervention.

Parameter	Standard diet	Casein-based HPD	Soy-based HPD
Number	6	6	6
Pulse rate (beats/min)	707 ± 38	718 ± 48	732 ± 34
Systolic blood pressure (mmHg)	129 ± 8	127 ± 7	129 ± 15
Plasma total protein (g/dL)	5.5 ± 0.4	5.1 ± 0.4	5.1 ± 0.3
Plasma total cholesterol (mg/dL)	156 ± 11	148 ± 18	129 ± 28
Plasma triglycerides (mg/dL)	192 ± 63	272 ± 134	270 ± 105

Values are presented as mean ± SD. No statistically significant differences were observed among the groups for any of the parameters listed.

Abbreviation: HPD, high-protein diet.

### Effects of casein-HPD and soy-HPD on visceral adipose tissue of obese diabetic KK-Ay mice

**FIGURE 2:**
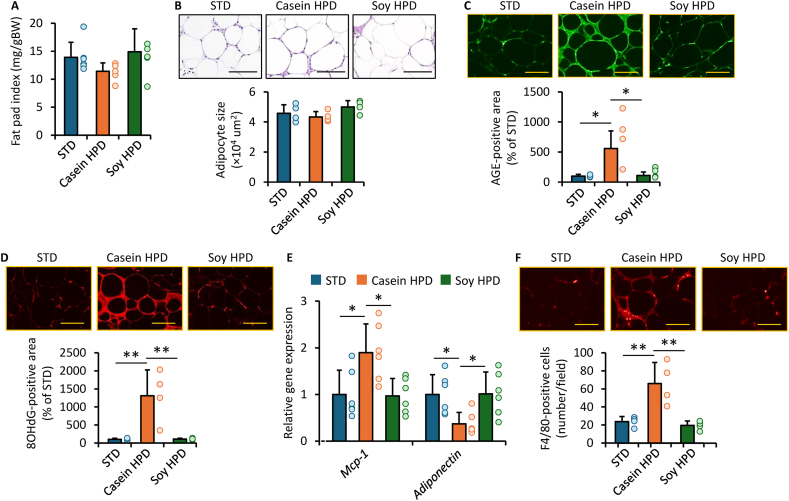
Effects of casein- and soy-based HPD on visceral adipose tissue of obese diabetic mice. (A) Epididymal fat pad index, expressed as the ratio of epididymal fat pad weight to body weight. (B) Adipocyte size. Upper panels show representative images stained with hematoxylin and eosin. Scale bars = 100 μm. (C, D) Glycer-AGE and 8-OHdG-positive areas. Upper panels show representative fluorescence images. Data are expressed as relative concentrations to the STD group. Scale bars = 100 μm. (E) Gene expression concentrations of target molecules. Expression levels were normalized to the internal control *18s rna* and are presented relative levels to the STD group. (F) Number of F4/80-positive cells. Upper panels show representative fluorescence images. Scale bars = 100 μm. (A, E) *n* = 6 per group; (B, C, D, F) *n* = 4 per group. ∗*P* < 0.05, ∗∗*P* < 0.01. 8-OHdG, 8-hydroxy-2′-deoxyguanosine; *18s rna*, 18S ribosomal RNA; AGE, advanced glycation end product; Glycer-AGE, glyceraldehyde-derived AGE; HPD, high-protein diet; *Mcp-1*, *monocyte chemotactic protein-1*; STD, standard diet.

Epididymal fat pad index and adipocyte size were comparable across the groups (Figure 2A, B). However, glycer-AGE accumulation concentrations in epididymal adipose tissue were significantly higher in mice fed the casein-HPD than in those fed the STD or soy-HPD (Figure 2C) and were positively correlated with HOMA-IR (*r* = 0.61, *P* = 0.047). Furthermore, this increase was accompanied by elevated concentrations of the oxidative stress marker 8-OHdG (Figure 2D) and by enhanced expression of the inflammatory gene *Mcp-1*, together with reduced expression of *adiponectin* (Figure 2E). Expression concentrations of *Mcp-1* and *adiponectin* were significantly correlated with HOMA-IR (*Mcp-1*: *r* = 0.61, *P* = 0.01; *adiponectin*: *r* = –0.52, *P* = 0.04). In contrast, Rage expression did not differ among the groups (data not shown). Moreover, the number of F4/80-positive macrophages was significantly higher in the adipose tissue of mice fed the casein-HPD than in those fed the STD or soy-HPD (Figure 2F).

### Effects of casein-HPD and soy-HPD on the kidney of obese diabetic KK-Ay mice

**FIGURE 3:**
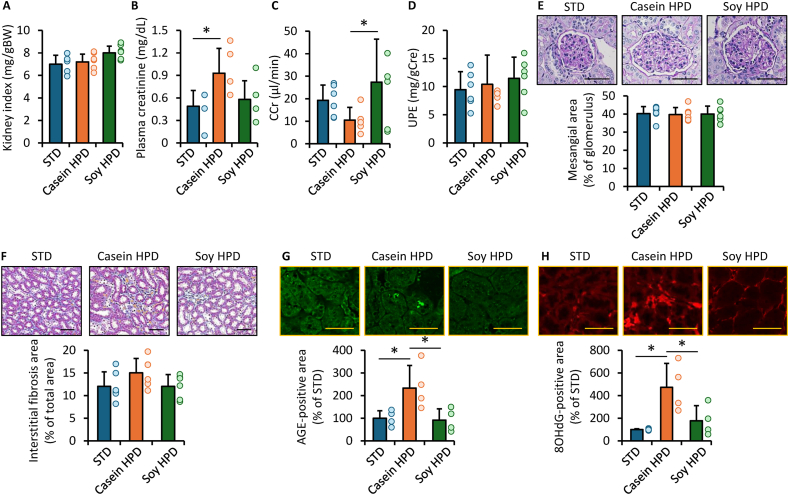
Effects of casein- and soy-based HPD on the kidneys of obese diabetic mice. (A) Kidney index, expressed as the ratio of kidney weight to body weight. (B) Plasma creatinine concentrations. (C) Creatinine clearance. (D) Urinary protein excretion (UPE). (E) Glomerular mesangial area. Upper panels show representative images stained with periodic acid–Schiff. Scale bars = 100 μm. (F) Interstitial fibrosis area. Upper panels show representative images stained with Masson’s trichrome. Scale bars = 100 μm. (G, H) Glycer-AGE and 8-OHdG-positive areas. Upper panels show representative fluorescence images. Data are expressed as relative levels to the STD group. Scale bars = 50 μm. (A–F) *n* = 6 per group, (G, H) *n* = 4 per group. ∗*P* < 0.05. 8-OHdG, 8-hydroxy-2′-deoxyguanosine; AGE, advanced glycation end product; CCr, creatinine clearance; Glycer-AGE, glyceraldehyde-derived AGE; HPD, high-protein diet; STD, standard diet.

As shown in Figure 3A–D, kidney index and urinary protein excretion were comparable among the groups. In contrast, plasma creatinine concentrations were significantly higher, and creatinine clearance was significantly lower in mice fed the casein-HPD compared with those fed the STD or soy-HPD. Histological findings are shown in Figure 3E and F. Glomerular mesangial area and interstitial fibrosis area did not differ among the 3 groups (Figure 3E and F). In contrast, renal glycer-AGE and 8-OHdG concentrations were significantly higher in mice fed the casein-HPD than in those fed the STD or soy-HPD (Figure 3G and H). Rage expression did not differ among the groups (data not shown).

### Glycer-AGE content in each diet

Glycer-AGE content in each diet was also quantified. Extraction efficiency was confirmed using glycer-AGEs prepared in our laboratory as previously described [23], yielding ∼40% recovery with either extraction buffer (before extraction: 4.0 mg/tube; after extraction: 1.6 mg/tube with 0.5% Triton X-100 and 1.6 mg/tube with 0.1% SDS). The glycer-AGE content in all diet samples was extremely low, measuring <0.1 μg/100 mg of diet.

## Discussion

In this study, we examined the effects of animal- and plant-based high-protein, low-carbohydrate diets on systemic metabolic parameters, glycer-AGE accumulation, and structural and functional alterations in adipose and renal tissues in KK-Ay mice. The proportions of protein and carbohydrate differed among the diets, whereas the fat content was kept constant across all groups. We found that the casein-HPD improved glycemic control without affecting body weight compared with the other diets; however, it significantly worsened insulin resistance. Although the casein-HPD did not alter visceral fat pad weight or adipocyte size, it markedly increased glycer-AGE accumulation in visceral adipose tissue, accompanied by elevated oxidative stress. Furthermore, the casein-HPD upregulated gene expression of *Mcp-1*, a key chemokine that drives macrophage recruitment [35], and correspondingly promoted macrophage accumulation in visceral adipose tissue. Chronic inflammation driven by macrophage accumulation, together with the resulting adipokine dysregulation, is well established as a central mechanism contributing to insulin resistance in type 2 diabetes [36]. Consistent with this concept, the casein-HPD reduced adiponectin expression, an adipocyte-derived factor that enhances insulin sensitivity [36]. In contrast, the soy-HPD did not evoke any of these adverse changes; rather, it lowered circulating glycer-AGE concentrations. Glycer-AGEs are known to increase *Mcp-1* expression in adipocytes through the induction of oxidative stress [[37], [38], [39]]. In the present study, the casein-HPD did not affect obesity or lipid profiles and even improved HbA1c, suggesting that these factors were unlikely to contribute to the observed changes in inflammatory and adipokine gene expression. Notably, increased *Mcp-1* and decreased *adiponectin* expression were both significantly correlated with HOMA-IR, indicating their relevance to insulin resistance in this model. Taken together, these findings suggest that a casein-HPD may exacerbate insulin resistance in obesity-associated diabetes, potentially by promoting glycer-AGE accumulation in visceral adipose tissue. This may lead to oxidative stress, macrophage infiltration, and subsequent adipocyte dysfunction, as illustrated in Supplemental Figure 1.

HPD has been shown in animal models to induce glomerular hyperfiltration through hemodynamic alterations, including afferent arteriolar dilation and increased intraglomerular pressure, ultimately promoting glomerulosclerosis and renal fibrosis in susceptible strains [40,41]. Although HPD can acutely increase glomerular filtration rates in humans, clinical evidence regarding HPD-induced kidney injury remains inconsistent, likely due to heterogeneity in study design and protein sources [[42], [43], [44], [45]]. Several studies have reported that animal-based, but not plant-based, HPDs exacerbate proteinuria and glomerulosclerosis in rodent models [10,11,15,16]; however, the mechanisms underlying these differences are not fully understood. In the present study, the casein-HPD–induced mild renal impairment, as reflected by increases in plasma creatinine and reductions in creatinine clearance, accompanied by glycer-AGE accumulation and elevated oxidative stress in the kidneys. Because body weight gain and visceral fat pad weights were comparable among the 3 groups, these renal functional changes are unlikely to be attributable to increased skeletal muscle mass or systemic anabolic effects induced by the casein-HPD. Notably, the casein-HPD did not alter proteinuria, glomerulosclerosis, or fibrosis in the kidney, nor did it influence systemic blood pressure. Therefore, the elevated plasma creatinine and reduced creatinine clearance may reflect local hemodynamic alterations, such as transient changes in glomerular filtration dynamics, rather than structural renal injury. Moreover, previous reports have shown that mouse models of diabetes typically develop only mild-to-moderate nephropathy compared with humans [41]. Thus, the duration of the dietary intervention in our study may have been insufficient to induce detectable structural changes.

In this study, we found that although insulin resistance was exacerbated in mice fed the casein-HPD, their HbA1c concentrations were significantly lower than those in mice fed the soy-HPD. HbA1c reflects long-term glycemic exposure, integrating both fasting and postprandial glycemia; however, fasting plasma glucose concentrations were unchanged in mice fed the casein-HPD. Therefore, we speculated that differences in postprandial glycemia may have contributed to the observed reduction in HbA1c. Energy intake was significantly lower in the casein-HPD group than in the soy-HPD group, which may partially account for a reduction in postprandial glycemia. In other words, the casein-HPD may attenuate postprandial hyperglycemia more effectively than the soy-HPD when carbohydrates are replaced by protein. Another potential explanation involves differences in energy expenditure, such as thermogenesis or basal metabolic rate. However, given that body weight gain and visceral fat pad weights were comparable among the 3 groups, these factors are unlikely to explain the observed differences in HbA1c concentrations.

In the present study, we found that the casein-HPD increased glycer-AGE accumulation in visceral adipose tissue and kidneys compared with the STD. Glycer-AGEs are formed through the nonenzymatic reaction of glyceraldehyde with primary amino groups on proteins, particularly arginine and lysine residues [46], and can arise from both endogenous formation and dietary sources [47]. In this study, however, glycer-AGE content in all diets was almost undetectable, consistent with our previous findings [48], indicating that the increased tissue glycer-AGE accumulation was not derived from dietary intake. A second possible explanation involves altered glyceraldehyde generation resulting from differences in amino acid composition. Glyceraldehyde is a highly reactive carbonyl species produced through multiple pathways, including glycolysis, glycation reactions, lipid peroxidation, and protein oxidation [49,50]. Casein and soy protein differ markedly in their amino acid profiles [51]. Among sulfur-containing amino acids, casein and soy protein contain higher concentrations of methionine and cystine, respectively [51]. Because methionine and cystine were supplemented to the casein-HPD and soy-HPD to equalize their total sulfur-containing amino acid content, this inherent difference is unlikely to explain the observed change in AGE accumulation. Notably, casein also contains higher concentrations of branched-chain amino acids, particularly leucine [51], which can enhance glycolytic flux and increase the intracellular NADH/NAD^+^ ratio [52,53]. These metabolic shifts may promote triose-phosphate degradation and glyceraldehyde formation, thereby potentially accelerating endogenous glyceraldehyde generation in mice fed the casein-HPD. In addition, casein is reported to have somewhat higher concentrations of amino acids with aromatic or heterocyclic rings, such as phenylalanine and histidine [51], which could contribute further to metabolic and oxidative stress under high-protein intake conditions [54,55]. In contrast, soy protein contains greater amounts of arginine and lysine [51] but induces less glycolytic stress, making it less likely to enhance glyceraldehyde production. A third potential factor is the difference in arginine and lysine contents in diets, which may influence glycer-AGE formation [46]. However, because soy protein contains higher concentrations of these amino acids than casein, this factor is unlikely to account for the greater glycer-AGE accumulation observed in casein-HPD-fed mice. Collectively, these findings suggest that differences in amino acid composition, particularly those affecting glycolytic flux and glyceraldehyde generation, may underlie the greater accumulation of glycer-AGEs observed in mice fed the casein-HPD.

There are several limitations to the present study. First, we used casein and soy as representative animal- and plant-based proteins, respectively [10,11,31]. Thus, it remains to be determined whether other types of animal- or plant-derived proteins exert effects similar to those observed here. Second, the fat content was kept constant across diets; therefore, the potential interactive effects of high-protein intake under high-fat conditions were not evaluated. Third, although the casein-HPD aggravated insulin resistance and was associated with increased macrophage infiltration and decreased adiponectin expression in epididymal adipose tissue, it remains unclear how and to what extent casein- and soy-HPDs differentially influence other fat depots, overall adiposity, and insulin-target organs such as the liver and skeletal muscle, as well as circulating factors that regulate systemic insulin sensitivity. Fourth, the sample size in the present study was relatively small, with substantial variability, which may have limited the ability to detect statistically significant differences for some parameters. Fifth, the mechanisms underlying the enhanced glycer-AGE accumulation in the casein-HPD group are still unclear. Experimental interventions using well-established antioxidants, such as N-acetylcysteine or tempol, or dietary restriction of branched-chain and aromatic amino acids may help determine whether oxidative stress-dependent metabolic alterations contribute causally to glycer-AGE production. Sixth, only male mice were employed in the present study to avoid potential confounding effects of hormonal fluctuations in females. Therefore, it remains uncertain whether sex differences may modify the metabolic effects of HPDs. Seventh, the HPDs used in this study were composed of ∼40% protein of total energy, which falls within the standard range commonly used in mechanistic rodent studies [32]. However, this protein content exceeds typical human intakes, which are generally around 15% to 20% of total energy and ∼25% to 30% even in HPDs. Therefore, caution is warranted when interpreting the present findings in clinical settings. Eighth, soy protein contains phytoestrogens, such as isoflavones, which may exert beneficial metabolic effects. A previous study demonstrated that supplementation with daidzein can improve glucose metabolism in KK-Ay mice [56]. However, phytoestrogen content was not quantified in the experimental diets in the present study, and the soy-HPD did not significantly affect body weight or glycemic parameters, including HbA1c, compared with the STD. Therefore, although phytoestrogens may contribute to metabolic regulation, their contribution to the observed metabolic differences between the casein- and soy-HPDs in this study remains unclear. Finally, this study employed a mouse model of obesity-associated diabetes [23,24]. Future studies are required to determine whether a casein-HPD also promotes glycer-AGE accumulation and/or induces insulin resistance under nonobese diabetic or nondiabetic conditions.

In conclusion, the present findings suggest that a casein-HPD was associated with greater insulin resistance in this model, concurrent with increased glycer-AGE accumulation in visceral adipose tissue, whereas the soy-HPD did not result in substantial differences compared with the STD. These observations suggest that the metabolic effects of HPDs may differ depending on the protein source.

## Author contributions

The authors’ responsibilities were as follows – YM, S-iY: designed the research; YS, YM, NO, MT, HY, TS, DT, MOgino, MOhara: conducted the research; YM, HY: provided essential materials, YS, YM: analyzed the data and performed the statistical analyses; YS, YM, S-iY: wrote the paper; YM: had primary responsibility for the final content; NO, MT, HY, TS, DT, MOgino, MOhara: contributed to paper revision; and all authors: read and approved the final manuscript.

## Data availability

Data described in the manuscript, code book, and analytic code will be made available on request pending application and approval.

## Funding

This study was funded by Grants-in-Aid for Scientific Research from the Ministry of Education, Culture, Sports, Science, and Technology of Japan (grant number: 24K17855).

## Conflict of interest

HY reports financial support was provided by Government of Japan Ministry of Education Culture Sports Science and Technology. YM reports a relationship with Boehringer Ingelheim GmbH that includes: funding grants. YM reports a relationship with Ono Pharmaceutical Co., Ltd. that includes: funding grants. If there are other authors, they declare that they have no known competing financial interests or personal relationships that could have appeared to influence the work reported in this paper.
