# Supplementary material for: Casein- and Soy-Based High-Protein Diets Differentially Affect Insulin Resistance and Adipose Tissue Advanced Glycation End Product Accumulation in Obese Diabetic Mice
Source: Curr Dev Nutr. 2026 Jan 27;10(3):107647. doi: 10.1016/j.cdnut.2026.107647 (PMC12945626; doi:10.1016/j.cdnut.2026.107647)
Supplement: multimedia component 1 [file mmc1.docx]

Supplemental Table 1. Composition of each experimental diet

|  | Standard diet | Casein-based  high-protein diet | Soy-based  high-protein diet |
| --- | --- | --- | --- |
| Energy density (kcal/g) | 4 | 4 | 4 |
| Macronutrient composition |  |  |  |
| Protein (% of total kcal) | 20.3 | 40.6 | 40.6 |
| Carbohydrate (% of total kcal) | 63.9 | 43.6 | 43.6 |
| Fat (% of total kcal) | 15.8 | 15.8 | 15.8 |
| Ingredient composition |  |  |  |
| Protein sources |  |  |  |
| Casein, lactic acid (g/kg) | 200 | 400 | 0 |
| Soy protein (g/kg) | 0 | 0 | 400 |
| L-Cystine (g/kg) | 3 | 6 | 0 |
| DL-Methionine (g/kg) | 0 | 0 | 6 |
| Carbohydrate sources |  |  |  |
| Corn starch (g/kg) | 397 | 194 | 194 |
| Maltodextrin (g/kg) | 132 | 132 | 132 |
| Sucrose (g/kg) | 100 | 100 | 100 |
| Cellulose (g/kg) | 50 | 50 | 50 |
| Fat sources |  |  |  |
| Soybean oil (g/kg) | 70 | 70 | 70 |
| Other ingredients |  |  |  |
| Tert-butylhydroquinone (g/kg) | 0.014 | 0.014 | 0.014 |
| Mineral mix S10022G (g/kg) | 35 | 35 | 35 |
| Vitamin mix V10037 (g/kg) | 10 | 10 | 10 |
| Choline Bitartrate (g/kg) | 2.5 | 2.5 | 0.25 |

Supplemental Figure 1. Proposed model of adipose tissue changes associated with a casein-based high-protein diet


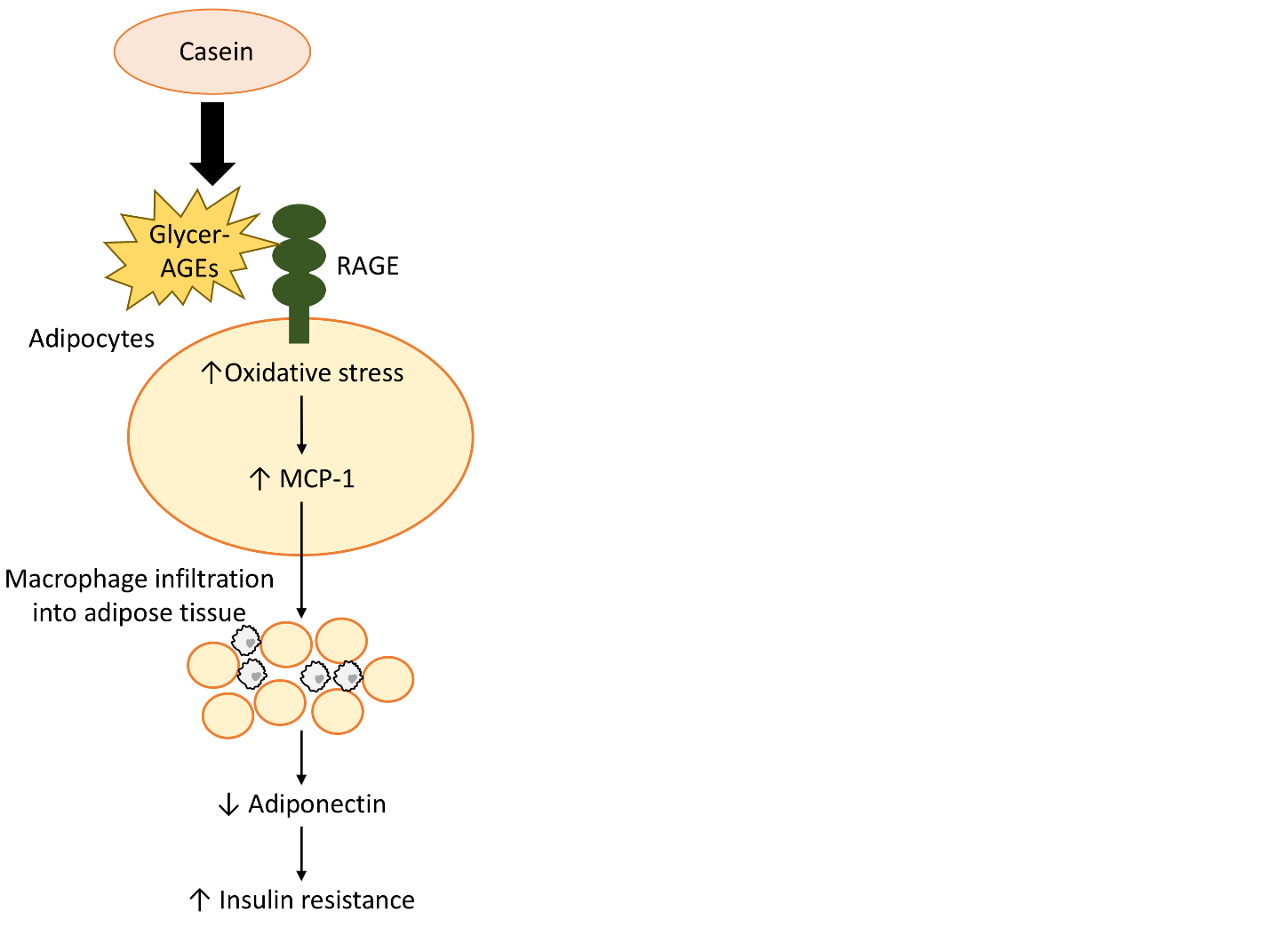


This schematic represents an associative model based on end-point measurements and does not imply a direct causal relationship.
